# Investigation of ciliary body inner diameter in Chinese myopic adults using ArcScan Insight® 100

**DOI:** 10.3389/fmed.2026.1898360

**Published:** 2026-09-21

**Authors:** Lei Luo, Xindi Wang, Qianrong Hu, Yan Cai, Linjuan Yang, Yu Zhang, Shimin Tan, Yimeng Fan, Zhao Liu, Qianyan Kang, Shengjian Mi

**Affiliations:** 1 Department of Ophthalmology, The First Affiliated Hospital of Xi’an Jiaotong University, Xi’an, Shaanxi, China; 2 Department of Ophthalmology, Xi’an Ninth Hospital, Xi’an, Shaanxi, China

**Keywords:** ArcScan Insight 100, Chinese adults, ciliary body inner diameter, ICL sizing, myopia

## Abstract

**Background:**

Ciliary body inner diameter (CBID) was recently reported as a new parameter for Implantable Collamer Lens (ICL) sizing. This study aimed to characterize CBID in Chinese myopic adults using the novel ArcScan Insight 100 very high-frequency ultrasound biomicroscope, identify its associated ocular anatomical factors, and assess its influence on Implantable Collamer Lens (ICL) size recommendations.

**Methods:**

In this cross-sectional study, 107 myopic eyes of 58 Chinese adults were enrolled. Anterior segment parameters, including CBID, were measured using ArcScan Insight 100. Linear mixed-effect models (LMMs) were fitted to identify determinants of CBID while accounting for inter-eye correlation. The relative contributions of independent variables were evaluated using part*R^2^*. ICL sizes calculated via the Reinstein formula (incorporating CBID) were compared with those from the STAAR OCOS formula (based on white-to-white diameter).

**Results:**

The mean CBID was 10.80 ± 0.48 (range 9.76 to 11.97) mm. Multivariate LMM analysis revealed that wider CBID was independently associated with younger age (*P* = 0.045), lower crystalline lens rise (CLR, *P* = 0.011), wider sulcus-to-sulcus diameter (STS, *P* < 0.001), and smaller STS lens rise (STSL, *P* = 0.017). Part*R^2^* analyses identified STS (part*R²* = 0.595) as the dominant contributor, explaining 59.5% of CBID variability. ICL sizes recommended by the Reinstein formula differed significantly from OCOS-based sizes (*F* = 4.672, *P* = 0.010), with 49% agreement.

**Conclusions:**

ArcScan Insight 100 allows direct measurement of CBID using a standardized protocol. In Chinese myopic adults, CBID was normally distributed and was mainly associated with STS. The Reinstein formula, which incorporates CBID, resulted in different ICL size recommendations for half of the eyes compared to the traditional WTW-based method. These findings demonstrate that incorporation of CBID can alter preoperative ICL size recommendations, though its direct effect on postoperative vault prediction requires future surgical validation.

## 1 Introduction

Ocular anterior segment parameters play important roles in Implantable Collamer Lens (ICL, STAAR Surgical) sizing, influencing the surgical safety. ICL sizing was initially based on the horizontal white-to-white diameter (WTW) (1). However, given that the ICL is ideally positioned in the ciliary sulcus, methods based on the sulcus-to-sulcus diameter (STS) were subsequently adopted to improve accuracy (2, 3). Nonetheless, studies indicate that, in clinical practice, a high proportion of ICL footplates rest on or insert into the ciliary body rather than the sulcus (4–6), which may contribute to the persistent incidence of suboptimal vault (distance between the ICL and natural crystalline lens, < 0.15 mm or > 1.0 mm) in 11.1% to 14.4% of eyes, even with STS-based sizing (2).

Advances in imaging technology have facilitated further refinement of ICL sizing. ArcScan Insight 100 (ArcScan, Inc., USA) is a very high frequency (VHF) ultrasound biomicroscope (UBM) that provides high-resolution anterior and posterior chamber imaging with good tissue penetration. Compared with conventional UBM, it acquires images in a seated position through a water-coupled disposable eyepiece, without direct probe contact with the cornea, which may reduce ocular deformation, improve patient tolerability, and support more standardized assessment of the ciliary sulcus, ciliary body, and other posterior chamber structures (7, 8). Since its introduction, it has been increasingly applied in anterior segment researches, especially the development and validation of ICL sizing methods. Utilizing measurements from this device, Reinstein developed a new ICL sizing formula that incorporates parameters including ciliary body inner diameter (CBID), which was more strongly correlated with postoperative vault than STS alone (6). This approach has been shown to improve vault prediction accuracy (6). Despite its potential, the distribution and determinants of CBID in the Chinese myopic population remain uncharacterized.

In this study, we used ArcScan Insight 100 (ArcScan, Inc., USA) to measure CBID and other ocular anterior segment parameters in Chinese myopic adults. We aimed to characterize the distribution of ArcScan-measured CBID, identify ocular anatomical factors associated with CBID, and evaluate whether incorporating CBID affects ICL size recommendations. These findings may provide anatomical data for the use of CBID in ICL sizing in Chinese myopic eyes.

## 2 Methods

### 2.1 Study design and subjects

This cross-sectional study enrolled a total of 107 myopic eyes of 58 subjects who underwent ophthalmological examinations in the Ophthalmology Refractive Surgery Center of the First Affiliated Hospital of Xi’an Jiaotong University in October 2025. The study was conducted in accordance with the ethical principles stated in the Declaration of Helsinki, and its ethics approval was granted by the Medical Ethics Committee of the First Affiliated Hospital of Xi’an Jiaotong University. All patients consented to the use of their data in subsequent studies.

The inclusion criteria included (1) age ≥ 18 years; (2) manifest refraction sphere ≤ −0.50 D; (3) anterior chamber depth (ACD) ≥ 2.6 mm. The exclusion criteria included (1) abnormal ophthalmic disease except refractive error, such as keratoconus, glaucoma and cataract; (2) history of previous intraocular or corneal surgeries; (3) complicating with severe diabetes, autoimmune or systemic connective tissue disease.

### 2.2 Measurements of ocular parameters

All subjects underwent a comprehensive ocular examination. The corneal topography and tomography (Sirius; CSO, Italy) was used to acquire the biometric parameters including the horizontal white-to-white diameter (WTW), ACD, mean of the flat and steep corneal curvatures, corneal thickness, and scotopic pupil diameter (SPD). Anterior and posterior chamber parameters, including ACD, anterior chamber angle (ACA), anterior chamber width (ACW), angle-to-angle diameter (ATA), crystalline lens rise (CLR), sulcus-to-sulcus diameter (STS), sulcus-to-sulcus lens rise (STSL) and ciliary body inner diameter (CBID), were measured using ArcScan Insight 100 (ArcScan, Inc., USA) very-high-frequency digital ultrasound robotic scanner following its practical guide (Figure 1). ACD was measured as the perpendicular distance from the corneal endothelium to the anterior surface of the crystalline lens along the central axis. ACA was measured as the angle formed by the posterior corneal surface and the anterior iris surface at the scleral spur, and the narrowest measured angle was recorded. ACW was defined as the scleral spur-to-scleral spur distance. ATA was measured between the deepest visible points of the angle recess on both sides. STS was measured between the bilateral ciliary sulci, with the sulcus defined as the end-point of the iris pigment epithelial echo. CBID was measured between the inner-most points of the ciliary body. CLR was defined as the perpendicular distance from the ATA plane to the anterior pole of the crystalline lens, and STSL as the perpendicular distance from the STS plane to the anterior lens surface. All caliper measurements were manually performed by one trained examiner and independently reviewed by a second trained examiner according to the standardized measurement protocol. Any discrepancies were resolved by consensus.

**Figure 1:**
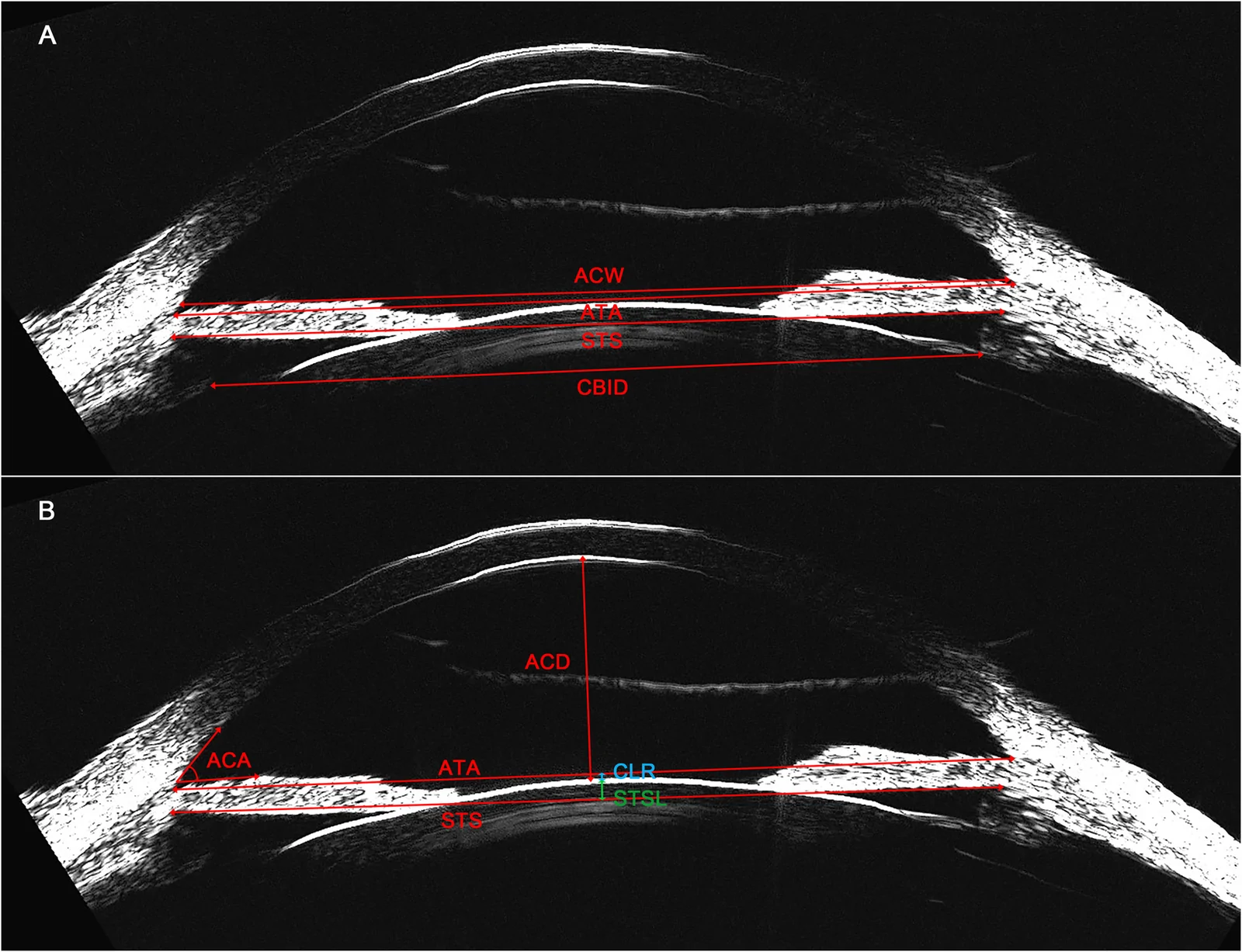
Parameters measured by Arcscan Insight 100. **(A)** Anterior chamber width (ACW), angle-to-angle (ATA) diameter, sulcus-to-sulcus (STS) diameter, ciliary body inner diameter (CBID); **(B)** anterior chamber depth (ACD), anterior chamber angle (ACA), the lens rise from the ATA plane described as crystalline lens rise (CLR), and the lens rise from the STS plane named STS lens rise (STSL).

For the measurements with Sirius (CSO, Italy), each subject was appropriately positioned on the chin rest, with both eyes kept at the same level and aligned with a central fixation light. Prior to each measurement, participants were asked to fully blink to ensure an even tear film distribution. Each examination was repeated three times. The scan with the highest image quality was chosen for analysis.

During scanning with ArcScan Insight 100 (ArcScan, Inc., USA), subjects were sitting upright, placing their chin into a headrest and the examined eye into an eye-cup, staring at the intermediate fixation light so that the pupil located in the center of the image. Their pupils were not dilated. The scan-set comprised seven cross-sectional B-scans at 0, 3, 6, 9, 351, 354, and 357 degrees. The image with highest quality was chosen to evaluate the horizontal biometry parameters.

### 2.3 Comparison of ICL sizing methods

The distribution of ICL sizes calculated using the Reinstein formula was compared with those obtained from STAAR OCOS website. The parameters measured by Sirius (CSO, Italy) were entered into STAAR OCOS website (https://evo-ocos.staarag.cn/ocoschina/?action=logoff) to acquire its recommended size and the maximum myopic lens sphere power. Subsequently, the maximum myopic lens sphere power, SPD, WTW and parameters measured utilizing ArcScan Insight 100 (ArcScan, Inc., USA) were entered into the ICL Sizing Calculator Version 1.1 (ICL Sizing Calculator Version 1.1) to calculate the predicted vault for each size (12.1 mm, 12.6 mm, 13.2 mm and 13.7 mm). The size with a predicted vault closest to 500 *μ*m was selected as the recommended size by Reinstein formula.

### 2.4 Statistical analysis

Statistical analysis was mainly conducted using SPSS 22.0 for Windows (IBM, Armonk, NY, USA). The normality of CBID was assessed using the Kolmogorov–Smirnov test. Linear mixed-effect models (LMMs) with subject as a random intercept were fitted to account for inter-eye correlation. Univariate and multivariate LMM analyses were performed with CBID as the dependent variable, while other ocular and general variables were included as independent variables. The relative contributions of independent variables were estimated by partitioning the *R^2^* values of each model using the function “part*R^2^*” from the “part*R^2^*” package of R software (v4.3.2; R Foundation for Statistical Computing, Vienna, Austria). The comparison of the distribution of ICL sizes calculated using the Reinstein formula with those obtained from STAAR OCOS website using generalized linear mixed model (GLMM). A two-sided *P*-value of less than 0.05 was considered statistically significant.

## 3 Results

The demographic and ocular characteristics are shown in Table 1. A total of 107 myopic eyes of 58 adult Chinese patients were included in this study. The age was 26.0 ± 6.4 years (range: 18 to 46 years). The MRSE was −5.11 ± 2.08 D (range: −10.25 to −0.75 D), with sphere of −4.58 ± 1.98 D (range: −9.50 to −0.75 D). The ACD was 3.06 ± 0.20 mm (range: 2.63 to 3.62 mm). The CBID was 10.80 ± 0.48 mm (range: 9.76 to 11.97 mm). The distribution of CBID among this study population is presented in Figure 2. It exhibits a normal distribution (*P* = 0.200).

**Table 1:** Demographic and ocular characteristics.

Parameter	Value
Number of eyes	107
Right eye (%)	57 (53.3%)
Male (%)	40 (37.4%)
Age (years)	26.0 ± 6.4 (18 to 46)
MRSE (D)	−5.11 ± 2.08 (−10.25 to −0.75)
Manifest refraction sphere (D)	−4.58 ± 1.98 (−9.50 to −0.75)
Manifest refraction cylinder (D)	−1.05 ± 0.69 (−3.00 to 0.00)
AL (mm)	25.43 ± 1.06 (23.50 to 28.41)
WTW (mm)	11.47 ± 0.35 (10.80 to 12.23)
SPD (mm)	6.59 ± 0.61 (4.93 to 7.85)
ACD (mm)	3.06 ± 0.20 (2.63 to 3.62)
ACA (degree)	46.89 ± 7.29 (32.31 to 72.41)
ACW (mm)	11.79 ± 0.33 (11.14 to 12.48)
ATA (mm)	11.62 ± 0.34 (10.91 to 12.69)
CLR (mm)	0.09 ± 0.13 (−0.289 to 0.468)
STS (mm)	11.50 ± 0.49 (10.30 to 12.69)
STSL (mm)	0.37 ± 0.13 (0.04 to 0.76)
CBID (mm)	10.80 ± 0.48 (9.76 to 11.97)

The values are presented as mean ± standard deviation (range) unless otherwise noted. SD, standard deviation; MRSE, manifest refraction spherical equivalent; D, diopter; CDVA, best-corrected distance visual acuity; AL, axial length; WTW, white-to-white distance; SPD, scotopic pupil diameter; ACD, anterior chamber depth; ACA, anterior chamber angle; ACW, anterior chamber width; ATA, angle-to-angle diameter; CLR, crystalline lens rise; STS, sulcus-to-sulcus diameter; STSL, sulcus-to-sulcus lens rise; CBID, ciliary body inner diameter.

**Figure 2:**
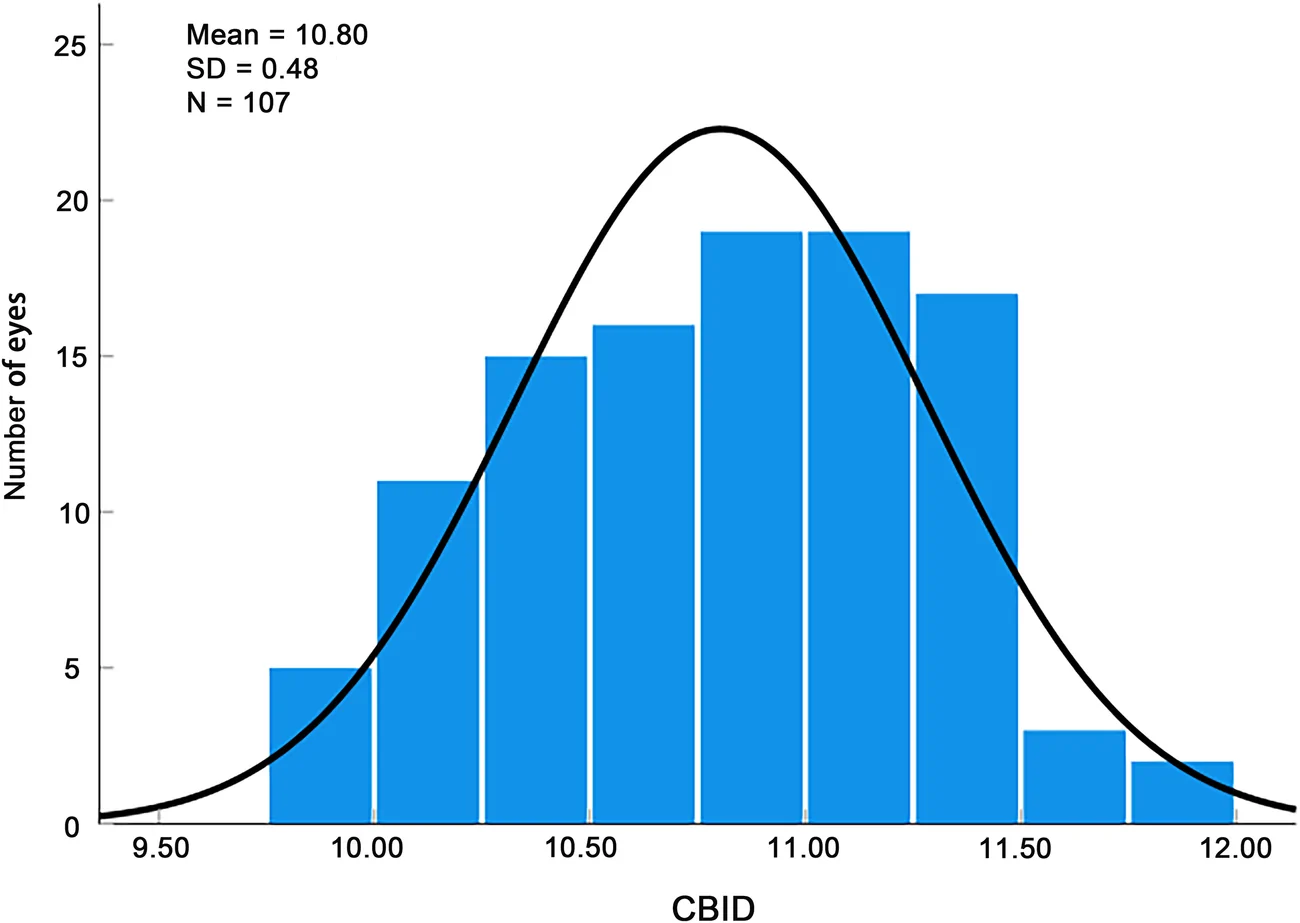
Distribution of ciliary body inner diameter (CBID) in the study population.

The results of LMM analyses for factors associated with CBID are summarized in Table 2. Univariate LMM analyses revealed that CBID was correlated with AL (marginal *R^2^* = 0.505, *P* < 0.001), ACD (marginal *R^2^* = 0.485, *P* < 0.001), ACW (marginal *R^2^* = 0.534, *P* < 0.001), ATA (marginal *R^2^* = 0.515, *P* < 0.001), STS (marginal *R^2^* = 0.791, *P* < 0.001), among which STS had the largest marginal *R^2^*. In multivariate LMM analyses, wider CBID was significantly correlated with younger age (*P* = 0.045), smaller CLR (*P* = 0.011), wider STS (*P* < 0.001) and smaller STSL (*P* = 0.017). We calculated variance inflation factors (VIFs) for all predictors in the multivariable LMM. All VIF values were below 5, indicating that collinearity was insufficient to substantially affect the stability of the multivariable model. Therefore, the resulting equation was CBID = 0.956 - 0.011 × age - 0.854 × CLR + 0.713 × STS - 0.782 × STSL. We further calculated their part*R^2^* and found that STS explained the largest proportion of the variability in CBID (part*R²* = 0.595), whereas the contributions of age (part*R²* = 0.024), CLR (part*R²* = 0.051), and STSL (part*R²* = 0.028).

**Table 2:** Linear mixed-effects model analyses for factors associated with ciliary body inner diameter.

Variable	Univariate LMM analyses			Multivariate LMM analyses		
	*β* (95% CI)	Marginal *R^2^*	*P*	*β* (95% CI)	*P*	VIF
Gender	0.097 (−0.141 to 0.335)	0.172	0.419	−0.057 (−0.199 to 0.084)	0.421	1.30
Age (years)	−0.003 (−0.021 to 0.015)	0.082	0.732	−0.011 (−0.022 to 0.000)	0.045*	1.30
AL (mm)	0.109 (0.090 to 0.271)	0.505	<0.001*	0.058 (−0.005 to 0.121)	0.072	1.31
WTW (mm)	0.236 (−0.078 to 0.550)	0.272	0.139	0.060 (−0.150 to 0.270)	0.571	1.49
ACD (mm)	0.925 (0.434 to 1.416)	0.485	<0.001*	0.247 (−0.137 to 0.631)	0.204	1.80
ACW (mm)	0.621 (0.359 to 0.883)	0.534	<0.001*	−0.253 (−0.634 to 0.127)	0.190	4.49
ATA (mm)	0.570 (0.322 to 0.817)	0.515	<0.001*	0.156 (−0.190 to 0.502)	0.372	4.97
CLR (mm)	−0.311 (−0.969 to 0.348)	0.148	0.351	−0.854 (−1.510 to −0.197)	0.011*	2.37
STS (mm)	0.698 (0.578 to 0.818)	0.791	<0.001*	0.713 (0.566 to 0.860)	<0.001*	1.79
STSL (mm)	−0.391 (−1.043 to 0.260)	0.187	0.236	−0.782 (−1.421 to −0.142)	0.017*	2.39

*Statistically significant (*P* < 0.05). LMM, linear mixed-effects model; VIF, variance inflation factors; AL, axial length; WTW, white-to-white distance; ACD, anterior chamber depth; ACW, anterior chamber width; ATA, angle-to-angle diameter; CLR, crystalline lens rise; STS, sulcus-to-sulcus diameter; STSL, sulcus-to-sulcus lens rise; CBID, ciliary body inner diameter.

Given the importance of CBID in the Reinstein ICL sizing formula, we further compared the ICL size recommended by the Reinstein formula and the OCOS formula (based on WTW). The distributions of the recommended size by the two formulas are shown in Figure 3A. Based on Reinstein formula, 15%, 70% and 15% of eyes were recommended to implant ICL of 12.1 mm, 12.6 mm and 13.2 mm, respectively. Whereas the rates were 18%, 51% and 31% respectively when calculating with OCOS formula. The distributions were significantly different (*F* = 4.672, *P* = 0.010). Compared to the size recommended by OCOS formula, the size recommended by Reinstein formula was same in 49% of eyes, smaller in 32% of eyes, and larger in 19% of eyes. The ICL size difference between the two formula is presented in Figure 3B.

**Figure 3:**
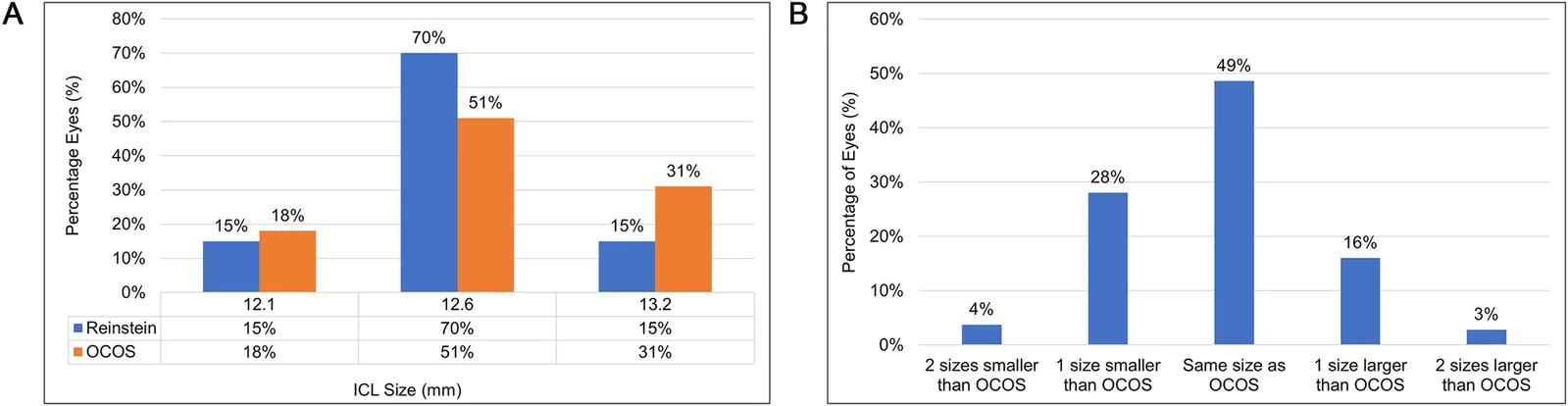
Comparison of implantable collamer lens (ICL) size recommended by the reinstein formula and OCOS formula. **(A)** Frequency distributions of ICL sizes recommended by the two formulas. **(B)** Difference in ICL size between the Reinstein formula and OCOS formula.

## 4 Discussion

Anterior segment parameters are essential for accurate ICL sizing, and their value depends on reliable measurement. Recently, Reinstein proposed a new ICL sizing method, firstly identifying the importance of CBID in predicting postoperative ICL vault (6). In this study, we used ArcScan Insight 100 (ArcScan, Inc., USA) to measure CBID and other anterior segment parameters in Chinese myopic adults. We found that CBID followed a normal distribution, was mainly associated with STS, and led to different ICL size recommendations in about half of eyes when incorporated into the Reinstein formula compared with the OCOS method.

We utilized a novel ultrasound device, ArcScan Insight 100 (ArcScan, Inc., USA), to acquire key parameters in our study. Previous measurements of ICL-sizing related posterior chamber parameters, such as STS, were mainly based on UBM and AS-OCT (1, 9). Traditional UBM is limited by the supine position and the requirements for direct contact with ocular surface, which may influence the results (10, 11). In contrast, the ArcScan Insight 100 (ArcScan, Inc., USA) allows imaging with the subject seated and without eyecup pressure on the eyeball, so that the acquired biometric parameters better reflect the physiologic anterior segment geometry. Moreover, the device is equipped with a broadband 20–60 MHz transducer, providing an axial resolution of 35 *μ*m and a lateral resolution of 65 μm, with a reported higher imaging accuracy than conventional ultrasound systems (10). Scanning along an arc approximating the anterior ocular surface curvature, it enables direct imaging and measurement of the entire anterior segment, including the anatomy behind the iris and scleral wall, such as the ciliary body and the choroidal space, which may be difficult to visualize completely with optical imaging techniques such as AS-OCT. Zhang et al (10), Zhu et al (12), and Huang et al (8) achieved direct *in vivo* analysis of ciliary muscle, anterior lens zonular length, and lens diameter, respectively, underscoring the device’s potential for posterior chamber imaging. Meanwhile, its repeatability for anterior segment parameters was favorable (all of ICC > 0.900), with CBID reaching an ICC of 0.984 (13). Al Farhan et al. also reported its good agreement with conventional UBM in ACD measurement (ICC = 0.990) (14). Clinically, accurate measurement of the posterior chamber, especially ciliary sulcus, is essential for the sizing and design of ICL and novel accommodative intraocular lenses, which rely on precise sulcus dimensions (15).

Ciliary body inner diameter is a posterior chamber parameter recently incorporated into ICL sizing formula (6). For WTW- or STS-based ICL sizing methods, several studies demonstrated a much more proportion of ICL haptics located at ciliary body position than directly at sulcus position, which significantly influenced the postoperative vault (4, 5). Subsequent investigations have reported significant associations between ciliary body-related anatomical parameters and haptic position or postoperative vault (16–19). Reinstein et al. found that STS lost significance for postoperative vault prediction after CBID was introduced into the ICL sizing formula (6). They proposed that a smaller CBID hints a smaller space for ICL, which makes the lens undertake more compression and thus lead to an increased vault. In this study, we investigated the anatomical characteristics of CBID and its impact on preoperative decision-making. It was defined, in accordance with the London Vision Clinic ArcScan Insight 100 Protocol and the Reinstein formula, as the distance between the inner-most points of the bilateral ciliary bodies, which is a protocol-defined, imaging-derived anatomical parameter rather than a direct measure of ciliary body biomechanical properties.

We explored the determinants of CBID, finding that STS showed the greatest and the most stable predictive value. STS and CBID are two anatomically adjacent posterior chamber parameters, providing an anatomical basis for their close association. Univariate LMM analyses demonstrated a highest correlation between CBID and STS, which was still significant in multivariate LMM analyses. STS explained 59.5% of the variability in CBID, indicating that the two parameters share more information related to the transverse dimensions of the posterior chamber. Nevertheless, STS alone did not fully account for CBID variability and therefore cannot be used to estimate CBID precisely. Besides, although ACW and ATA were significantly associated with CBID in the univariate analyses, none retained significance in the multivariate model. WTW remain nonsignificant correlation with CBID during both univariate and multivariate analyses. Given their anatomical interrelation with STS, collinearity may affect the stability and precision of their individual regression coefficients. We therefore calculated their VIF values to assess the multicollinearity. All VIFs were below 5, suggesting no substantial multicollinearity or clinically important inflation of standard errors in the multivariable model. STS meanwhile showed the strongest and most consistently supported association with CBID. Therefore, anterior chamber parameters such as ACW and WTW cannot be used to determine CBID size precisely. Direct measurement of the posterior chamber is preferable when CBID is required.

In addition to STS, CBID was associated with multiple ocular anatomical parameters. Age, CLR and STSL showed significant associations with CBID in multivariate analysis. CLR and STSL are lens-height parameters related to lens position and lens thickness. Greater CLR indicates a thicker lens or a lens with more pronounced lens anterior protrusion (20), which would push the ciliary process forward and change the width of CBID. In addition, as age increases, the lens undergoes progressive thickening (21), and the lens proptosis becomes more pronounced, which in turn exerts biomechanical stress on the ciliary processes and narrows the CBID (22). Meanwhile, the effect of aging on the ciliary body has also been reported (22, 23). Nevertheless, the contributions are relatively limited. The potential effects of lens position, aging, and detailed ciliary body morphology require confirmation in larger studies.

The potential implication of CBID was further explored by comparing a CBID-incorporated Reinstein formula with the WTW-based STAAR Surgical proprietary formula (STAAR OCOS formula). The distribution of ICL sizes recommended by the two methods was significantly different and agreed in 49% of eyes. Thirty-two percent of eyes were recommended to receive 1 size smaller ICLs than that recommended by STAAR OCOS website. These results were similar to Reinstein’s study (6) (same in 40%, 1 size smaller in 34%) and Izdebska’s study (24) (same in 50%, 1 size smaller in 27%). The STAAR OCOS formula is primarily based on WTW and ACD, which do not directly measure the posterior chamber structure. As for Reinstein formula, the included anatomical parameters mainly consisted of CBID, STSL and SPD, wherein CBID exhibits the largest predictive effect (6). The weak correlation between CBID and WTW may partly account for the discrepancy in size recommendations between the two formulas. However, this discrepancy cannot be attributed solely to these parameters, as the Reinstein formula also incorporates lenticular and pupillary effects. Additionally, Reinstein’s study (6) and Izdebska’s study (24) demonstrated that the distribution of ICL sizes recommended by Reinstein formula was more similar to that calculated utilizing sulcus-based sizing methods [Dougherty (3) and Kojima (2)], which was consistent with the relatively high correlation between CBID and STS. These findings indicate that incorporating posterior chamber parameters can affect preoperative ICL size selection. However, further studies incorporating postoperative vault outcomes are needed to determine the specific effects of CBID in ICL sizing for Chinese myopic eyes.

This study has several limitations. First, the sample size was relatively small and the range of age was restricted. Second, the parameters were selected mainly according to the Reinstein sizing framework (6), and more detailed ciliary body morphological parameters were not included. Third, although the device enables direct imaging of posterior chamber structures, the relevant parameters still require manual measurement. Fourth, the study was cross-sectional and did not include postoperative vault validation; therefore, the sizing comparison should be considered a preoperative anatomical and decision-impact analysis. Prospective studies with postoperative vault and haptic position outcomes are required to determine whether CBID provides incremental predictive value beyond STS and whether this information improves ICL-sizing accuracy in Chinese myopic eyes.

## 5 Conclusions

To the best of our knowledge, this is the first study to characterize CBID in Chinese myopic adults using ArcScan Insight 100, which enables direct measurement of CBID using a standardized protocol. We found that CBID showed a normal distribution and was associated with multiple ocular anatomical parameters, with STS as the strongest predictor. Incorporating CBID into the Reinstein formula changed the recommended ICL size in about half of eyes compared with the WTW-based OCOS method, but whether it improves ICL sizing accuracy requires postoperative validation.

## Data Availability

The raw data supporting the conclusions of this article will be made available by the authors, without undue reservation.
